# Antimicrobial Activity of Metabolites Secreted by the Endophytic Bacterium *Frateuria defendens*

**DOI:** 10.3390/plants9010072

**Published:** 2020-01-06

**Authors:** Alaa Naama-Amar, Shani Gitman, Nofar Shoshana, Ofir Bahar, Vered Naor, Einat Zchori-Fein, Lilach Iasur-Kruh

**Affiliations:** 1 Department of Biotechnology Engineering, ORT Braude College of Engineering, Karmiel 2161002, Israel; 2 Department of Entomology, Agricultural Research Organization, Newe Ya’ar Research Center, Bet Shearim 3657800, Israel; 3 Department of Plant Pathology and Weed Research, Agricultural Research Organization, Volcani Center, Rishon LeZion 7534509, Israel; 4 Shamir Research Institute, Qatsrin 1290000, Israel

**Keywords:** *Frateuria defendens*, yellows disease, endophytic bacteria, Phytoplasma, 4-quinolinecarboxaldehyde, 5-hydroxymethyl-2-furaldehyde, Metabolites

## Abstract

*Candidatus* Phytoplasma, the causative agent of yellows disease, inflicts substantial damage on several hundred plant species including perennials and annual plants. The endophytic bacterium *Frateuria defendens* reduces the symptoms of yellows disease in a number of agricultural crops. One possible mode of action is that the bacterium secretes antimicrobial metabolites. To test this hypothesis, the substances secreted by the endophyte during 10 days of growth in an artificial medium were identified by GC-MS (gas chromatography–mass spectrometry). Synthetic analogues to these substances were then used on periwinkle, a nurse culture plant infected by phytoplasma. Phytoplasma quantities were evaluated by quantitative PCR, and disease symptoms were monitored and recorded. It was found that specific compounds identified by the biochemical analysis caused a significant reduction in both the titer of phytoplasma and the disease symptoms in periwinkle when compared to untreated infected plants. Further research is required to examine the potential of these compounds as an effective treatment against yellows disease.

## 1. Introduction

Bacteria-inhabiting plants and their surroundings secrete natural products that can play a significant role in developing sustainable agriculture [1]. In particular, endophytes (microorganisms that inhabit the plant inner tissues without causing any harm) produce bioactive compounds with chemical structures that have been optimized by evolution to enhance plant fitness including protection from different phytopathogens [2]. Hence, metabolites secreted by endophytic bacteria can potentially be integrated into disease management strategies for improving the control of plant diseases. Diseases inflicted by phloem-inhabiting bacteria from the genus *Candidatus* Phytoplasma (Acholeplasmatales; Acholeplasmataceae) (hereafter referred to as phytoplasma) are vectored by insects of the Hemiptera order and harm various plants including perennial and annual crops [3]. Wine grapes are a high-value crop that suffers from substantial damage caused by Ca. Phytoplasma solani and Ca. Phytoplasma vitis, which inflict the Bois Noir and Flavescence dorée diseases, respectively [4,5,6]. Current management approaches of phytoplasma diseases rely mainly on limiting the population of the insect vector by chemical pesticide application. However, in many cases such as Bois Noir disease, the polyphagous nature of the vector dramatically reduces the efficiency of this approach [5,7]. Another effective treatment is the liquid trunk injection of antibiotics (mainly oxytetracycline), but the usage of antibiotics as a pest management strategy is expensive and prohibited in most countries [8]. Therefore, effective but safe antimicrobial agents are needed to control severe phytoplasma diseases.

Recently, we have shown that the endophytic bacterium *Frateuria defendens* (Xanthomonadales, Rhodanobacteraceae) significantly reduces yellows disease symptoms in phytoplasma-infected grapevine plants. Hence, it was proposed that *F. defendens* could be considered as a beneficial bacterium, and may serve as a biocontrol agent against phytoplasma in grapevines [9]. *Frateuria defendens* (formerly referred to as *Dyella*-like bacterium or DLB; [10]), is a Gram-negative bacterium isolated from the planthopper *Hyalesthes obsoletus* (Hemiptera, Cixiidae), the insect vector of Bois Noir disease [11]. Since both phytoplasma and *F. defendens* are found in the planthopper, interaction between them inside the insect is possible and needs to be further examined. However, unlike phytoplasma that inhabits the insect midgut, hemocoel, and salivary glands, *F. defendens* was found only in the insect gut, suggesting that it was not vertically transmitted, but rather acquired from the plant [11]. Indeed, upon foliage application, *F. defendens* was shown to penetrate the leaf surface and inhabit the vascular tissues of various plant crops including grapevine, orange, pomelo, cotton, cucumber, and carrot, with no apparent phytotoxic effect [12]. Further research showed that a supernatant of *F. defendens* culture inhibits the culture growth of *Spiroplasma melliferum* (Entomoplasmatales, Spiroplasmataceae), which is a cultivable model of phytopathogenic Mollicutes [9]; the secretion of metabolites, which directly inhibit phytoplasma growth, was suggested as one possible mechanism by which *F. defendens* may reduce yellows symptoms.

Since phytoplasma cannot be grown in an axenic culture, a nurse culture system infected with this bacterium is essential for assessing various means of control strategies in planta [7]. Periwinkle (*Catharanthus roseus*; Gentianales, Apocynaceae) can serve as a model nurse culture system for a wide variety of phytoplasma types that cause disease in multiple host plants. Phytoplasma infection of periwinkle results in very distinctive symptoms such as the narrowing and yellowing of leaves and the development of green flowers (virescence) [13].

In light of the previous results, we aimed to: (1) determine whether metabolites secreted by *F. defendens* could potentially have a protective role against phytoplasma; (2) identify active *F. defendens*-secreted metabolites; and (3) test synthetic analogous of *F. defendens* metabolites on a bacterial model *in vitro* and *in planta*.

## 2. Results and Discussion

Antibiosis, the secretion of pathogen-inhibiting substances, is one of several possible disease-suppression mechanisms of bacterial biocontrol agents (bBCA) [14,15,16]. In previous studies, *F. defendens* was isolated, characterized, and identified as a potential bBCA of phytoplasma, with antibiosis as a possible mode of action [9]. Numerous bio-active metabolites that are toxic to pathogens are secreted by different bBCA such as hydrogen cyanide, phenazines, pyrrolnitrin, and pyoluteorin [17] and can be used as effective control agents with or without the secreting bacterium [18,19]. Therefore, the aim of the current study was to investigate the effect of secreted metabolites from *F. defendens* on phytoplasma titer and yellows disease symptoms in infected periwinkle and to identify the active metabolites.

### 2.1. The Effect of F. defendens Filtrate on phytoplasma Symptoms in Periwinkle

To examine the bio-control activity of substances originating in *F. defendens* filtrate, the roots of *ex vitro* periwinkles infected with phytoplasma from the alfalfa witch’s broom type (hereafter, infected-plantlets) were trimmed and submerged in the filtrate. Although this method does not simulate the conditions that can be applied in the field, it secures the entrance of various substances into the plantlets. The treated-infected plantlets exhibited a significantly higher survival rate (83%) compared with untreated ones (25%) (chi square = 7.1034, *P* = 0.007). No significant difference in the survival rate was observed between healthy *ex vitro* plantlets (hereafter, H-plantlets) and infected-plantlets treated with the filtrate (chi square = 0.545., *P* = 0.46) (Table 1). As no damage signs to the leaves were observed for both treated and un-treated H-plantlets, it was concluded that under the tested conditions, the filtrate was not phytotoxic. Quantitative real time-PCR (qPCR) results confirmed the presence of phytoplasma in all infected-plantlets, whereas the pathogen could not be detected in the tissue of H-plantlets (average Ct value equals 28 and 33, respectively, *t*-test, *P* < 0.001). Phytoplasma Ct values were not significantly different between the infected-plantlets regardless of whether they were treated or not with the *F. defendens* filtrate (Table 1), meaning that the filtrate reduced the plantlets’ mortality, but did not affect the phytoplasma concentration in the plant. These results suggest that the filtrate of *F. defendens* contains compounds that affect plant morphology without causing direct damage to the pathogen. Since phytoplasma has been reported to interfere with the hormonal network of its host plant [20], it is possible that *F. defendens* secretes substances that enhance plant growth and balance the periwinkle disrupted system, though this possibility warrants further examination.

### 2.2. Identification of Potentially Active F. defendens Metabolites

**Figure 1:**
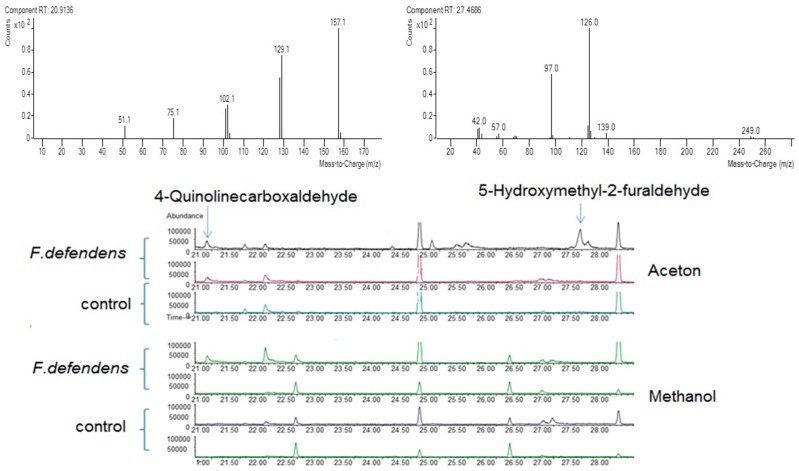
Identification of *F. defendens*-secreted metabolites by gas chromatography–mass spectrometry (GC-MS) analysis. The *F. defendens* culture supernatant was fractionated using different solvents. Fractions showing spiroplasma inhibition activity were analyzed by GC-MS. The lower section shows the gas chromatography spectrum of substances extracted by acetone and methanol (two-sequel fractions for each extract) from the filtrate of *F. defendens* or from the sterile control medium. Arrows indicate the dominant compounds found only in the fraction containing the bacteria. The upper section shows the mass spectra of these compounds: 4-quinolinecarboxaldehyde (**left**) and 5-hydroxymethyl-2-furaldehyde (**right**).

To identify the active antimicrobial compounds secreted by *F. defendens*, culture supernatants were fractionated using polar and hydrophobic solvents, and each fraction was applied separately to *Spiroplasma melliferum* cultures and tested for its inhibition activity. Out of the ten fractions tested, only three inhibited *S. melliferum* growth: two sequel fractions extracted by acetone and one extracted by methanol. No inhibition could be detected by fractions extracted using methyl *tert*-butyl ether (MTBE), chloroform, and hexane as well as the second fraction extracted by methanol. Similarly, *S. melliferum* growth was not inhibited by any of the control fractions (sterile medium without bacteria extracted by the same solvents). Gas chromatography–mass spectrometry (GC-MS) analysis of the active fractions revealed 11 different compounds (see Table S1); however, only two of them were unique to the active fractions and absent in the inactive fractions: 4-quinolinecarboxaldehyde (QC; Base peak area 24,942) and 5-hydroxymethyl-2-furaldehyde (HMF; Base peak area 113,950), which was dominant in the first acetone active fraction (Figure 1).

HMF is an organic compound that consists of a furan ring containing both aldehyde and alcohol functional groups [21,22]. HMF was detected in secretions of *Idriella bolleyi*, a soil fungus that serves as a biocontrol agent against the wheat fungal pathogen *Gaeumannomyces graminis*. In addition, this compound was found to inhibit several plant pathogenic bacteria (*Xanthomonas axonopodis, Pectobacterium carotovorum, P. crhysanthemi*, and *Erwinia amylovora*) as well as human pathogens (*Bacillus cereus*, *Proteus mirabilis*, and *Escherichia coli*) *in vitro* [23].

QC is a derivative of quinoline, which is a heterocyclic aromatic organic compound with a strong odor. This compound was identified as the active ingredient of the *Ruta chalepensis* (Sapindales, Rutaceae) extract, and shown to inhibit *Clostridium perfringens* and *E. coli* [24]. Other derivatives of this group were found to be effective against phytopathogenic fungi and are used as fungicides in agriculture (http://lilab.ecust.edu.cn/ptid/compound/browse.html?class=105). To the best of our knowledge, no record exists regarding the effect of HMF and/or QC on phloem-restricted pathogens.

### 2.3. The Effect of 4-quinolinecarboxaldehyde (QC) and 5-hydroxymethyl-2-furaldehyde (HMF) on Growth of S. melliferum and Other Bacteria

**Figure 2:**
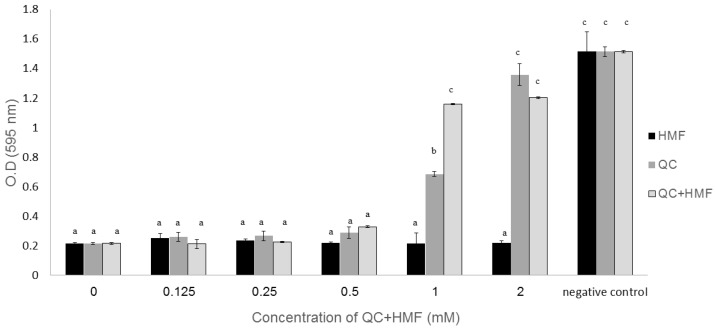
The effect of 4-quinolinecarboxaldehyde (QC) and 5-hydroxymethyl-2-furaldehyde (HMF) on spiroplasma growth. Each compound was applied to a spiroplasma culture (5 µL containing approximately 10E8 cells/mL and 150 µL medium) in different concentrations, separately and combined. Low OD represents a change in color from red to yellow, indicating culture growth; high OD represents no change in color, indicating no growth. OD reads were taken at 594 nm by an ELISA plate reader three days post inoculation and incubation at 28 °C. Sterile medium containing 20 μL of each of the fractions was used as a negative control. Each treatment was performed in five repeats. Different letters represent significant differences in growth between treatments (*t*-test, *p* < 0.05). Standard error bars are shown.

To determine whether the two compounds identified in the active filtrate affect *S. melliferum* growth, we tested purified and commercially available QC and HMF molecules using the *S. melliferum* inhibition assay. QC at 1 and 2 mM concentrations significantly inhibited the growth of *S. melliferum* compared to the control (*t*-test, *P* < 0.05) (Figure 2). HMF was not effective when applied alone in the tested concentrations (0.125–2 mM), but a combination of QC and HMF at 1 mM significantly increased the inhibitory efficacy on spiroplasma compared to QC alone (*t*-test, *P* < 0.05) (Figure 2).

**Figure 3:**
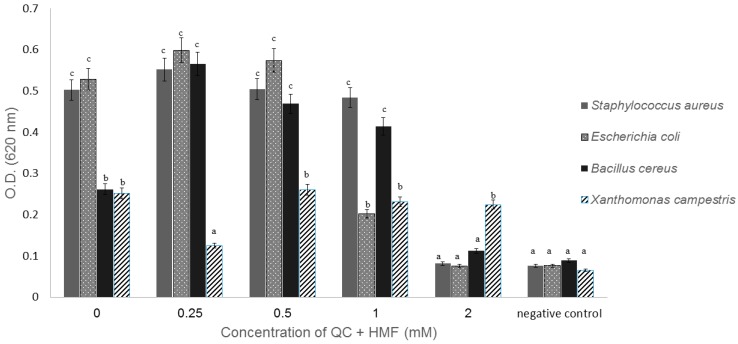
The effect of 4-quinolinecarboxaldehyde (QC) and 5-hydroxymethyl-2-furaldehyde (HMF) on the growth of several Gram-negative and Gram-positive bacteria; different concentrations of QC and HMF were applied to bacterial cultures (5 µL containing approximately 10E8 cells/ mL and 150 µL NB medium). The amount of each bacterium, represented by turbidity, was read by an ELISA plate reader 24 h post inoculation and incubation at 28 °C. A sterile medium was used as a negative control. Each treatment was performed in five repeats. Different letters represent significant differences in growth between treatments (*t*-test, *p* < 0.05). Standard error bars are shown.

Furthermore, this combination affected the growth of other species tested including Gram-positive (*Staphylococcus aureus* and *Bacillus cereus*) and Gram-negative (*Escherichia coli* and *Xanthomonas campestris*) bacteria: *B. cereus* growth was induced in the presence of 0.25–1 mM QC and HMF concentrations, but was inhibited at 2 mM. The minimum inhibitory concentration (MIC) of *S. aureus* and *E. coli* was 2 mM and 1 mM, respectively, while *X. campestris* was significantly reduced at 0.25 mM but not by any of the other concentrations tested (Figure 3). The fact that this combination of compounds inhibited both Gram-positive, Mollicutes and Gram-negative bacteria suggests that the operating mechanism is quite broad. Several bacteria from different orders (Actinomycetales, Rhizobiales, Burkholderiales, Pseudomonadales, etc.) have enzymes that are able to transform HMF to 2-furoic acid and further to 2-oxoglutarate, resulting in detoxification of this compound [25], but the presence of these enzymes in spiroplasma was never examined. If spiroplasma is able to detoxify HMF, perhaps QC inhibits the activity of these transformation enzymes, enhancing the inhibitory effect of HMF and causing a synergistic effect. Indeed, the use of combinations of various natural products has received much attention due to the potentially beneficial synergistic effects, which include a reduction in the pathogens’ resistance rates and a reduction in the infection rate of diseases that are difficult to control [26].

### 2.4. QC and HMF Phytotoxic Effect

Phytotoxicity tests of the QC and HMF combination on the H-plantlets revealed that a concentration of 5 mM is lethal (all plantlets died within three days), while all plantlets survived the exposure to 1 and 2 mM solutions (Table 2), but the latter caused leaf chlorosis within seven days post treatment.

However, applying 1 mM of QC and HMF on **phytoplasma-infected** plantlets was lethal (Table 2).

Application of these compounds on **mature** periwinkle plants showed a phytotoxic effect at 10 and 20 mM concentrations when drenched, and 20 mM when sprayed on the leaves. No effects on the survival rate and morphology of the mature plants were detected when a 5 mM concentration was applied by drenching or spraying (Table 2). These results indicate that the efficiency of the QC and HMF combination was 5 (5 = nonphytotoxic effect/1 = MIC). The fact that the phytotoxic concentration in H-plantlets was lower than in mature plants can be explained by their sensitive metabolic condition after being transferred from *in vitro* conditions [27].

Notably, the effect of the filtrate on the infected plantlets was beneficial, as it reduced disease symptoms without causing any phytotoxic effect (see results in the section above). Previous studies have shown that the application of a single bBCA metabolite was less effective than the application of the bBCA supernatant as a whole [19]. This is most likely due to the presence of several other active metabolites secreted by bBCA, which may have an additive or synergistic effect [19].

### 2.5. The Effect of QC and HMF on Phytoplasma in Periwinkle

The effect of HMF and QC on mature periwinkle plants grafted with phytoplasma-infected plants scions (hereafter, grafted-periwinkle) was further examined. Grafted periwinkle were either drenched or sprayed with the 5 mM solution of QC and HMF. Untreated grafted-periwinkle served as a positive control while un-grafted plants served as negative controls. qPCR was used to assess the phytoplasma titer in the grafted-plants. Four weeks post-grafting, the phytoplasma quantity was similar in all samples to the un-grafted periwinkles controls. Twelve weeks post-grafting, phytoplasma was detected in the leaves of untreated grafted-periwinkle and sprayed grafted-periwinkle at similar levels (R = 4.1 × 10^3^ and 5.8 × 10^3^, respectively, *P* > 0.05). In contrast, in grafted-periwinkle drenched with QC and HMF, the phytoplasma levels remained similar to the un-grafted periwinkle controls (Table 3). Furthermore, after 12 weeks, the **drenched** grafted-periwinkle showed no phytoplasma symptoms, while 50% of the un-treated grafted-periwinkle exhibited typical phytoplasma symptoms in the form of green flowers and yellow leaves. Grafted-periwinkle **sprayed** with HMF and QC developed disease symptoms at the same level as untreated grafted plants (Table 3).

The differences in effectiveness between drenching and spraying application could perhaps be explained by the waxy leaves of periwinkle plants, which shed most of the spray application, while the ability of the compounds to systemically move through the roots to the vascular tissues may be more effective. Indeed, it is known that the pesticide application technique may affect treatment efficiency [28]. Even though drenching intact plants is less aggressive than the submergence of trimmed roots, QC and HMF delivered by this technique significantly reduced phytoplasma infection.

A possible explanation for the fact that the crude extract affected only phytoplasma symptoms but not its titer, while QC and HMF affected both the phytoplasma titer and symptoms, is that the phytoplasma-infected plantlets contained a high titer of phytoplasma while the grafted periwinkles had an initial lower titer transmitted from the graft, and therefore the phytopathogen was more susceptible to inhibition.

In conclusion, phloem-limited plant pathogenic bacteria such as *Phytoplasma*, *Spiroplasma*, and *Liberibacter* pose a major threat to a list of agricultural crops [12].

While various effective bactericides/bacteriostatic compounds have been identified, delivering them to the site where the bacteria reside (i.e., the phloem sieve elements), is a challenging task [5,6,7]. This study shows that metabolites secreted from the beneficial bacterium *F. defendens* can serve as a basis for the development of effective chemical formulations against phloem-inhabiting pathogenic bacteria. Studies of the protective ability of secreted metabolites of *F. defendens* against phytoplasma need to be conducted in other plants.

## 3. Materials and Methods

### 3.1. Bacteria Origin and Growth

*Frateuria defendens* strain DHoT, originally isolated from *H. obsoletus* in 2011 [10,11], was used in all experiments. The strain type DHoT is deposited in both the NCCB (The Netherlands Culture Collection of Bacteria) (100648T) and DSM (German Collection of Microorganisms and Cell Cultures) (106169T) [10].

To conduct experiments with *F. defendens* supernatants, the bacterium was streaked on crystal violet (CV) plate (sucrose at 66 g/L, sorbitol at 10 g/L, Luria Broth (Sigma-Aldrich, St. Louis, MO, USA) at 2 g/L, 0.1% [wt/vol] CV, and agar at 15 g/L), and then a single colony was transferred to a 5 mL spiroplasma medium [29] starter. The whole 5-mL starter was transferred into the spiroplasma medium, in a 500-mL Erlenmeyer containing 200 mL of defined without antibiotics, and cultures were grown for 10 days at 28 °C with shaking. The culture was then centrifuged for 10 min at 4000 rpm to pellet bacterial cells and the supernatant was filtered through a 0.22 µm pore filter (vacuum bottle filters, JET BIOFIL, Elgin, UK). The pellet was plated on a CV plate to ensure pure culture, and the filtrate was diluted 1:2 with sterile double distilled water (DDW). The filtrate was immediately used in order to examine its effect on phytoplasma symptoms in periwinkle.

*Spiroplasma melliferum BC3*, obtained from Naor’s Laboratory, served as a model bacterium for *in vitro* examination, as previously detailed by [11].

### 3.2. Fractionation of F. defendens Supernatant

To identify metabolites with potential activity, *F. defendens* was grown for ten days in 500 mL of medium containing 6 g/L Luria Broth, 2 g/L K_2_HPO_4_, and 0.5 g/L KH_2_PO_4_ for ten days at 28 °C with shaking [11]. The culture was centrifuged at 8000 rpm for 5 min, and the supernatant was extracted twice with 500 mL MTBE, according to [30]. Briefly, the organic phase was evaporated to dryness using a gentle stream of nitrogen, and the dry extract was re-suspended with MTBE. This crude extract was separated to fractions by a 4 mL column containing glass wool, sand, and 600 mg silica (Sigma-Aldrich, St. Louis, MO, USA) using the following different solvents: methanol, acetone, MTBE, chloroform, and hexane (each solvent produced two sequel fractions). The same extraction procedure was performed on a sterile medium as the control. Each fraction was re-evaporated to dryness using a gentle stream of nitrogen, and the dried substances were re-suspended with 1 mL of ethanol. The procedure above was performed using glassware in order to avoid volatile contamination.

### 3.3. Periwinkle Origin and Growth Conditions

Periwinkle (*Catharanthus roseus*, Apocynaceae) was chosen as a model plant for in planta phytoplasma inhibition and disease suppression experiments as it is a common host in which phytoplasma can replicate and cause specific disease symptoms [13,31]. Each mature periwinkle plant was grown in 0.5 L pots with soil (EN12580, Tuf Merom Golan, Merom Golan, Israel) in a growth chamber under a long-day photoperiod (16 h light) at 28 °C.

Healthy plantlets (in short: H-plantlets) or, plantlets infected with phytoplasma alfalfa witch’s broom (in short: infected-plantlets) were sub-cultured routinely *in vitro* every ca. 60 days at the Naor laboratory in MS agar with sucrose and growth factors (MS 4.33 g/L, myoinositol 100 mg/L, thiamine-HCl 0.4 mg/L, benzyl adenine 450 μg/L (2 μM), naphthaleneacetic acid 376 μg/L (2 μM), sucrose 3% and agar-agar 5.7 g/L at pH 5.7) in 23–25 °C under a long-day photoperiod (16 h light) (Shamir Institute, Katzrin, Israel; [9]. The presence of phytoplasma was monitored by nested-PCR using primers P1/P7 followed by U3/U5 or R16F2n/R162r [32,33].

### 3.4. Grafted Periwinkle

Since the infected-plantlets were sensitive to the application of identified GC-MS compounds, as shown by the reduced viability (see Table 2), we further examined the compounds’ effect on mature periwinkle plants grafted with phytoplasma-infected plants (grafted-periwinkle): Mature (two-month-old) periwinkle plants were obtained from a commercial nursery (Galil Plants, Rakefet, Israel). Periwinkle plants were graft-inoculated with scions collected from one source of periwinkle plant, which was infected with the phytoplasma group 16SrII-D in the Bahar Laboratory. The grafting technique that was followed has been previously described [13]. To enable transmission of the pathogenic bacteria into the vascular system, the grafted shoots were kept on the plant for one week and then removed to prevent continuous inoculation from scion to rootstock. Growth conditions for grafted periwinkles were the same as those described for the plantlets (see above).

### 3.5. Phytoplasma Quantification

Phytoplasma titer was measured by quantitative real-time PCR (qPCR) in all samples. DNA from 100 mg tissue of periwinkle was extracted by a commercial kit according to the manufacturer’s instructions (Norgen plant/fungi DNA isolation kit, Norgen Biotek Crop., Thorold, Canada). The extracted DNA served as a template for the qPCR assay using the SYBR green method (Fast SYBR Green, Applied Biosystems, MA, USA), with the specific primer pair for ’phytoplasma Phytovinca_R-GCCTCTGGTGTTCCTCCATA, Phytovinca_F-TGGTGTAATGGCAACGCTTA) designed in the current study. Each reaction tube contained 3 µL DDW, 5 µL Fast SYBR Green Master Mix (ThermoFisher Scientific), 0.5 µL of each primer (5 pmol), and 1 µL DNA. In order to examine the quality and quantity of DNA, each sample was also quantified for the ubiquitin gene of periwinkle using specific primers (UBQvinca_L-GGAAGGACTTTGGCTGACT, UBQvinca_R-AGCAGGCAGCAGAACAATTT) [34].

### 3.6. The Effect of F. defendens Filtrate on Phytoplasma Symptoms in Periwinkle

The roots of the phytoplasma-infected plantlets were washed from agar residues in which they were grown, trimmed with scissors, and submerged in the filtrate of *F. defendens* or in a sterile control medium for 24 h, and then planted in plastic-covered 0.5 L pots with soil. Each of the two treatments consisted of 12 plantlets (replicates). H-plantlets, which underwent the same procedure (washed, trimmed, and submerged in filtrate or in a sterile control medium; six for each treatment) served as a negative control. Viability and morphology of all plantlets were evaluated once a week, but significant differences were observed only after 20 days.

### 3.7. Identification of Potentially Active F. defendens Metabolites

The effect of each fraction obtained from the *F. defendens* supernatant (see fractionation of *F. defendens* supernatant section) on *S. melliferum* growth was examined in Eppendorf tubes containing 20 μL of each fraction with one mL of *S. melliferum* medium supplemented with phenol red as an indicator for culture growth, as was previously described [29,35]. As a control, *S. melliferum* was grown in a sterile medium. Twenty μL of each of the fractions was added to tubes containing the sterile medium in order to rule out the possibility of contamination. Each treatment was conducted in triplicate (three independently inoculated Eppendorf tubes).

Only fractions that showed an inhibitory effect on *S. melliferum* growth (see Results) were chosen for further analysis. To identify the specific active compounds, a 1 µL aliquot of the sample from different phase extracts was injected into a GC-MSD apparatus (6890N/5973N Agilent Technologies, Santa Clara, CA, USA), as previously reported with small modifications [30]: Helium (constant pressure 15.2 psi) was used as a carrier gas. The injector temperature was 250 °C, set for a splitless injection. The oven was set to 50 °C for 1 min, and then the temperature was increased to 260 °C at a rate of 5 °C/min. The detector temperature was 28 °C. The mass range was recorded from 41 to 350 m/z, with an electron energy of 70 eV. A mixture of straight-chain alkanes (C7–C23) was injected into the column under the above-mentioned conditions for the determination of retention indices. The GC-MS spectrum profiles were analyzed using the ChemStation software. The identification of the volatiles was conducted by comparing their retention indices with those in the literature and by comparing their spectral data with the standard or with the W9N08 and HPCH2205 GC-MS libraries.

### 3.8. The Effect of QC and HMF on the Growth of S. melliferum

Two compounds, 4-quinolinecarboxaldehyde (QC) and 5-hydroxymethyl-2-furaldehyde (HMF), were identified in the active fraction extracts of the *F. defendens* supernatant. One hundred mM stock solutions (dissolved in EtOH absolute) of synthetic analogs of these compounds (QC- catalog number 176966, and HMF- catalog number H40807; Sigma Aldrich, Rehovot, Israel) were used to test their potential inhibition. The spiroplasma growth inhibition test was performed in 96-well plates: each well contained 150 µL of spiroplasma medium, 5 μL *S. melliferum* cells (obtained from frozen spiroplasma stock containing ~10^8^ cells/mL), and varying concentrations (0.125–2 mM) of each compound separately or in combination. The plates were incubated for three days at 28 °C. *S. melliferum* growing in a sterile broth with 5 µL ethanol served as a positive control, and a sterile medium containing the compounds without spiroplasma was used as a negative control. The plates’ readings were scored at 594 nm by an ELISA plate reader (MULTISKAN EX, Thermo Electron Corporation, Waltham, MA, USA). Changes in color from red to yellow indicated *S. melliferum* growth. Each treatment included five replicates (five independently inoculated wells in the plate). This experiment was conducted twice with similar results.

The combination of QC and HMF produced the highest inhibition effect, and thus it was used in the rest of the experiments.

### 3.9. The Effect of QC and HMF on Growth of Different Bacteria

To assess the potential of the combination of QC and HMF to inhibit the growth of other bacteria, the following species were used: *Staphylococcus aureus, Escherichia coli, Bacillus cereus* (Biological Industries, Bet HaEmek, Israel)**, and *Xanthomonas campestris pv. campestris 33913* (obtained from Bahar Laboratory). Each of these bacteria was isolated on a nutrient agar plate (Sigma Aldrich, Rehovot, Israel), and then a single colony was transferred to a 5 mL starter of nutrient broth (Sigma Aldrich, Rehovot, Israel). The effect of the QC and HMF combination was examined in 96-well plates: each well contained 150 µL of nutrient broth, 5 μL each of bacterial culture, and varying concentrations (0.125–2 mM) of both QC and HMF. The plates were incubated for 24 h at 37 °C. Each bacterial species grown in sterile broth served as a positive control and a sterile medium containing the compounds without the bacteria was used as a negative control. Turbidity indicated bacterial growth was measured as the optical density of the cultures at a 620 nm wavelength with an ELISA plate reader (MULTISKAN EX, Thermo Electron Corporation, Thermo Electron Corporation, Waltham, MA, USA). Each treatment included five replicates (five independently inoculated wells in the plate). This experiment was conducted twice with similar results.

### 3.10. QC and HMF Phytotoxic Effect

The phytotoxic effect of QC and HMF on *ex vitro* plantlet and mature periwinkle plants was examined. H-plantlet roots were submerged in different concentrations of the QC and HMF combination (5, 2, and 1 mM) for 24 h (in triplicate: three different plantlets for each concentration), before planting in the controlled conditions as described above. Mature periwinkles were drenched or sprayed with 10 mL of QC and HMF (20, 10, 5, and 1 mM). Each treatment was conducted in four replicates. The phytotoxic effect was examined by monitoring plant morphology (changes in leaf color) and viability for seven days.

### 3.11. The Effect of QC and HMF on Phytoplasma

The roots of 18 infected-plantlets and 10 H-plantlets were submerged in 1 mM QC and HMF (the highest non-toxic concentration **for *ex vitro* plantlets**) overnight, potted, and grown in controlled conditions. Morphology and viability of the plants as well as phytoplasma titer were evaluated every week.

Grafted-periwinkle (plants grafted with a phytoplasma-infected scion for a week) were drenched (12 mL) or sprayed (until runoff) with 5 mM QC and HMF (the highest non-toxic concentration **for mature periwinkles**) at weekly intervals for three months, starting one day after the grafting. Each treatment contained six replicates (plants), and five healthy periwinkle plants served as a negative control. Plant morphology and viability were evaluated every two weeks until the end of the experiments. Phytoplasma concentrations were evaluated by qPCR one and three months after the grafting.

### 3.12. Statistical Analyses

Descriptive statistics, analysis of variance, and Chi square tests were performed with SPSS software. Chi square was used in order to test significant differences in plant survival rates. *P* values of less than 0.05 were considered to be statistically significant.

ΔΔCt calculation was performed by first normalizing the phytoplasma gene with the plant housekeeping gene, ubiquitin. Second, the results, obtained after 12 weeks of each treatment (un-grafted periwinkle, untreated-grafted periwinkle, drenched and sprayed grafted periwinkle) were compared with the results of each treatment at the first time point, as explained in [36]. In other words, each treatment was compared to itself, three months vs. the start point, thereby revealing the relative rise in phytoplasma.

## Figures and Tables

**Table 1:** The effect of the *F. defendens* filtrate on the viability and phytoplasma titer (estimated by qPCR) of *ex vitro* periwinkle plantlets 20 days post treatment.

Treatment	Plantlets	Phytoplasma-16S rRNA Gene Ct *	Periwinkle-Ubiquitin Gene Ct *	% Survival (n)
None	Healthy	33.1 ± 0.4 ^a^	21.8 ± 0.4 ^a^	100 (6) ^a^
	Infected	28.1 ± 1 ^b^	21.6 ± 0.3 ^a^	25 (12) ^b^
*F. defendens* filtrate	Healthy	33.2 ± 1 ^a^	21.6 ± 0.4 ^a^	100 (6) ^a^
	Infected	28.2 ± 0.7 ^b^	21.3 ± 0.5 ^a^	83 (14) ^a^

* Values represent average Ct of all examined plantlets (including the ones that did not survived) ± SE. Different letters represent significant differences (*P* < 0.05).

**Table 2:** Phytotoxicity: Periwinkle survival rates as affected by 4-quinolinecarboxaldehyde (QC) and 5-hydroxymethyl-2-furaldehyde (HMF) application. *Ex vitro* plantlets were submerged in the solution for 24 h before planting, while mature plants were drenched or sprayed. Survival rates were examined a week post-treatment.

Periwinkle (n)	QC and HMF Concentration (mM)	Survival (%)		Leaves Color
*Ex vitro* H-plantlets (3)	0	100 *^,a^		green
	1	100 ^a^		green
	2	66 ^a^		yellow
	5	0 ^b^		
*Ex vitro* phytoplasma-infected plantlets (12)	1	0 ^b^		
		**Drenched**	**Sprayed**	
Mature plants (4)	0	100 ^a^	100 ^a^	green
	5	100 ^a^	100 ^a^	green
	10	25 ^b^	100 ^a^	green
	20	0 ^b^	25 ^b^	green
Grafted-mature periwinkle (6)	5	100 ^a^	100 ^a^	green

* Chi test > 4.5, *P* < 0.05 in comparison to untreated plants. Different letters represent significant differences in survival between treatments (*t*-test, *p* < 0.05).

**Table 3:** The effect of different application techniques of 4-quinolinecarboxaldehyde (QC) and 5-hydroxymethyl-2-furaldehyde (HMF) on phytoplasma symptoms and titer in grafted with phytoplasma infected plants and un-grafted periwinkles 12 weeks post grafting. Each treatment was performed in six repeats.

	Un-Grafted Periwinkle	Grafted Untreated Periwinkle	Grafted Periwinkle—Drenched	Grafted Periwinkle—Sprayed
**Symptomatic plants (%)**	0 ^a,^*	50 ^b^	0 ^a^	50 ^b^
R (2^^−ΔΔCt^) **	ND	4.1 × 10 ^3^	ND	5.8 × 10^3^

* Chi test > 4, *P* = 0.045 in comparison to the untreated plants; Different letters represent significant differences. ** see calculation in M & M; ND—below detection level.

## Supplementary Materials

The following are available online at https://www.mdpi.com/2223-7747/9/1/72/s1, Table S1: Different compounds identified from the active fractions and control fractions of *F. defendens* supernatant.
