# Supplementary material for: Antimicrobial Activity of Metabolites Secreted by the Endophytic Bacterium Frateuria defendens
Source: Plants (Basel). 2020 Jan 6;9(1):72. doi: 10.3390/plants9010072 (PMC7020481; doi:10.3390/plants9010072)
Supplement: Supplementary file 1 [file plants-09-00072-s001.pdf]

Table S1: Different compounds identified from the active fractions and control fractions of *F. defendens* supernatant

Compound	Component RT	Compound Name	Match Factor	Formula
RT1	8.3	8.69 Limonene	68	C10H16
RT2	10.5	2-Phenylethanol	91	C8H10O
RT3	17.1	4-methylquinazoline	92	C9H8N2
RT4	18.4	Benzeneacetamide	90	C8H9NO
RT5	20.9	4-Quinolinecarboxaldehyde	90	C10H7NO
RT6	24.9	Phosphoric acid tributyl ester	94	C12H27O4P
RT7	25.5	2-methylindole-3-carbaldehyde	92	C10H19NO
RT8	27.5	2-Furaldehyde, 5-(hydroxymethyl)-	87	C6H6O3
RT9	29.0	29.57 Silphiperfolan-6-beta-ol<7-epi->	30	C15H26O
RT10	29.9	1,3,2-Oxathiaborolan-2-amine, N,N-diethyl-	89	C6H14BNOS
RT11	32.2	2-tert-Butyl-1,4-dioxaspiro[4.5]deca-6,9-dien-8-one	82	C12H16O3
